# Genotypic Analyses of Shiga Toxin-Producing *Escherichia coli* O157 and Non-O157 Recovered from Feces of Domestic Animals on Rural Farms in Mexico

**DOI:** 10.1371/journal.pone.0051565

**Published:** 2012-12-10

**Authors:** Bianca A. Amézquita-López, Beatriz Quiñones, Michael B. Cooley, Josefina León-Félix, Nohelia Castro-del Campo, Robert E. Mandrell, Maribel Jiménez, Cristóbal Chaidez

**Affiliations:** 1 Centro de Investigación en Alimentación y Desarrollo, Culiacán, Sinaloa, México; 2 U.S. Department of Agriculture/Agricultural Research Service, Produce Safety and Microbiology Research Unit, Western Regional Research Center, Albany, California, United States of America; 3 Facultad de Ciencias Químico Biológicas, Universidad Autónoma de Sinaloa, Culiacán, Sinaloa, México; U. S. Salinity Lab, United States of America

## Abstract

Shiga toxin-producing *Escherichia coli* (STEC) are zoonotic enteric pathogens associated with human gastroenteritis worldwide. Cattle and small ruminants are important animal reservoirs of STEC. The present study investigated animal reservoirs for STEC in small rural farms in the Culiacan Valley, an important agricultural region located in Northwest Mexico. A total of 240 fecal samples from domestic animals were collected from five sampling sites in the Culiacan Valley and were subjected to an enrichment protocol followed by either direct plating or immunomagnetic separation before plating on selective media. Serotype O157:H7 isolates with the virulence genes *stx2*, *eae*, and *ehxA* were identified in 40% (26/65) of the recovered isolates from cattle, sheep and chicken feces. Pulse-field gel electrophoresis (PFGE) analysis grouped most O157:H7 isolates into two clusters with 98.6% homology. The use of multiple-locus variable-number tandem repeat analysis (MLVA) differentiated isolates that were indistinguishable by PFGE. Analysis of the allelic diversity of MLVA loci suggested that the O157:H7 isolates from this region were highly related. In contrast to O157:H7 isolates, a greater genotypic diversity was observed in the non-O157 isolates, resulting in 23 PFGE types and 14 MLVA types. The relevant non-O157 serotypes O8:H19, O75:H8, O111:H8 and O146:H21 represented 35.4% (23/65) of the recovered isolates. In particular, 18.5% (12/65) of all the isolates were serotype O75:H8, which was the most variable serotype by both PFGE and MLVA. The non-O157 isolates were predominantly recovered from sheep and were identified to harbor either one or two *stx* genes. Most non-O157 isolates were *ehxA*-positive (86.5%, 32/37) but only 10.8% (4/37) harbored *eae*. These findings indicate that zoonotic STEC with genotypes associated with human illness are present in animals on small farms within rural communities in the Culiacan Valley and emphasize the need for the development of control measures to decrease risks associated with zoonotic STEC.

## Introduction

Shiga toxin-producing *Escherichia coli* (STEC) is a group of food- and water-borne pathogens that are known to cause human gastrointestinal illnesses with diverse clinical spectra, ranging from watery and bloody diarrhea to hemorrhagic colitis [1], [2]. In some cases, disease symptoms result in the life-threatening, hemolytic uremic syndrome (HUS), and it is thought that Shiga toxins (Stx1 and Stx2) are the key virulence factors contributing to the development of HUS. Although more than 200 different serotypes of STEC have been isolated, O157:H7 has been the serotype most commonly associated with HUS in North America. Recent epidemiological studies have recognized additional non-O157 serogroups, including O26, O45, O91, O103, O104, O111, O113, O121, and O145, among STEC strains that were linked to severe human disease in the United States, Europe and countries of Latin America [3]–[7].

Epidemiological studies have shown that not all STEC strains producing Stx are clinically relevant. Thus, it has been proposed that accessory STEC genes may also contribute to human disease [4], [8], [9]. For example, a well-characterized adhesin gene is *eae* that codes for the intimin protein, implicated in attachment to the intestinal epithelial cells prior to lesion formation [10], [11]. Many of the strains implicated in bloody diarrhea and HUS in humans are *eae*-positive; therefore, *eae* is recognized as an important risk factor for HUS [1], [12]. Additionally, enterohemolysin, expressed by the *ehxA* gene, liberates hemoglobin from the red blood cells during infection and has been linked to severe disease symptoms [13]–[15]. Consequently, *E. coli* strains, recovered from animal reservoirs and harboring *stx*, *eae*, and/or *ehxA* genes, are thought to represent a subpopulation of STEC strains that may pose a higher risk to human health [16], [17].

STEC strains have been isolated from a variety of animals, and cattle are considered to be the major reservoir for STEC strains [1], [18]–[20]. However, recent evidence has indicated that small domestic ruminants, including sheep and goats, are also key reservoirs of STEC [21]–[26]. In particular, sheep and their products have been documented as reservoirs for STECs that belong to a diverse set of non-O157 serogroups (O26, O91, O115, O128, and O130) and that harbor genes encoding key virulence factors that have been implicated in human disease [27]–[33]. STEC strains can also be carried by other domestic and wild animals, such as cats, dogs, rodents, deer, birds, feral pigs, chickens, and insects [18], [34], [35].

A few recent studies have examined the prevalence of O157 and non-O157 STEC strains in products derived from animals or in animal fecal samples at various locations in Mexico [36]–[39]. In particular, a greater prevalence of *E. coli* O157 was found in swine feces (2.1%) than cattle feces (1.25%), recovered from eight different locations throughout Central Mexico [36]. Furthermore, the presence of O157 as well as non-O157 on beef carcasses was confirmed at slaughter plants in Northeast and Western Mexico [38], [39]. Surveillance studies yielded recovery of non-O157 STEC strains in a large proportion of ready-to-eat meals in Mexico City [40], suggesting that non-O157 could be a potential source of infection in humans. Thus, additional studies about isolation, sources, and prevalence of STEC are needed in other agricultural locations in Mexico.

To determine relevant animal reservoirs for toxigenic *E. coli* in Mexico, the present study employed a protocol for the selective enrichment of STEC from feces of domestic animals found on small rural farms within the Culiacan Valley. The Culiacan Valley, located in Northwest Mexico, has large and fertile agricultural fields and a successful packaging industry for horticultural commodities (tomatoes, cucumbers, and bell peppers) [41]. The small rural farms, sampled in the present study, were located in rural communities where the primary purpose of raising livestock is for local consumption [42]. The recovered STEC isolates were further examined for O- and H-antigens and several important virulence factors. Furthermore, the genetic relatedness of the isolates was analyzed by the genotyping methods pulse-field gel electrophoresis (PFGE) and multiple-locus variable-number tandem repeat analysis (MLVA) to obtain a better understanding of the geographical distribution and relevant sources of zoonotic STEC in the Culiacan Valley.

## Results

### Isolation of STEC from Feces of Farm Animals in the Culiacan Valley

**Figure 1:**
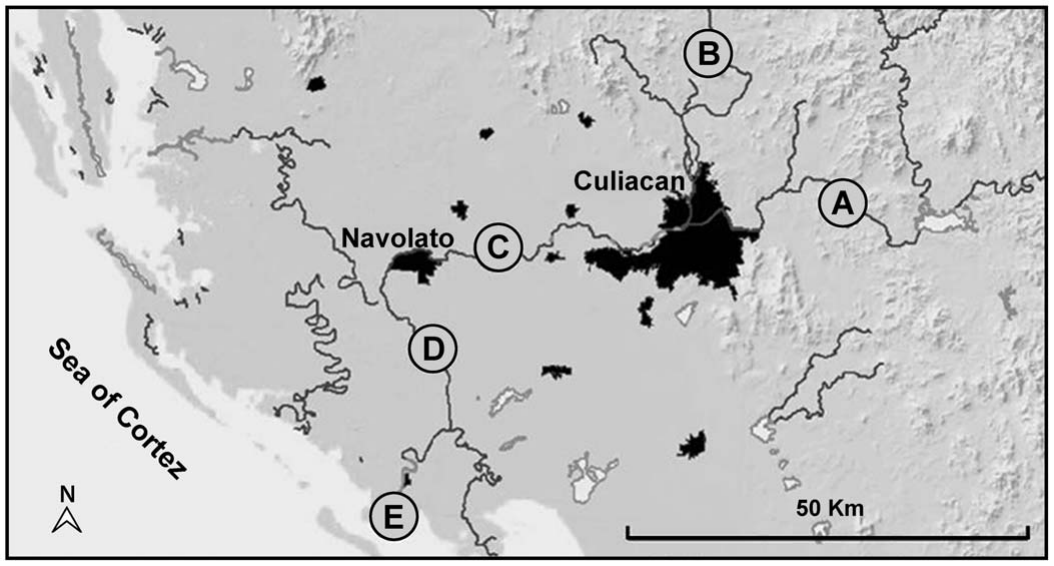
Sampling sites in the Culiacan Valley in Northwest Mexico. Map of the five sampling sites, Jotagua (A), Agua Caliente (B), Cofradia de Navolato (C), Iraguato (D), and El Castillo (E) that were selected to be 14–38 km apart and to represent the study area in the Culiacan Valley, Sinaloa, Mexico. Dark areas in the map indicate urban zones with more than 20,000 inhabitants. Scale bar corresponds to 50 km.

The aim of the present study was to identify both O157 and non-O157 STEC recovered from feces of domestic farm animals in the Culiacan Valley, a region in Northwest Mexico. Fecal samples were recovered from small farms at five sampling sites in the Culiacan Valley, each located in close proximity to rivers used for irrigation (Figure 1). During the first six months of the sampling (July to December 2008), enrichment broths were prepared from a total of 120 fecal samples from all sampling sites and were plated directly onto the indicator media (see Materials and Methods). Presumptive STEC colonies were recovered from 5% (6/120) of the fecal samples after plating on both indicator media (Table 1). Sampling sites C, D, and E had 8.3% (either 1/12 or 2/24) positive samples when compared to 4.2% (1/24) for sampling site B. Presumptive STEC colonies were predominantly detected in 11.1% (4/36) of the sheep fecal samples and, secondly, in 3.3% (2/60) of the cattle fecal samples (Table 1). No positive samples were obtained from sampling site A after plating directly from the enrichment broths onto the two indicator media.

**Table 1:** Proportion of fecal samples positive for presumptive STEC.

Method^1^	Source	Number of positive samples/Number of fecal samples tested per sampling site^2^					
		Site A	Site B	Site C	Site D	Site E	Total
Direct plating	Cattle	0/12	1/12	0/12	1/12	0/12	2/60
	Chicken	0/12	0/12	NA^3^	NA	NA	0/24
	Sheep	0/12	NA	2/12	NA	2/12	4/36
	Total	0/36	1/24	2/24	1/12	2/24	6/120
IMS	Cattle	0/12	0/12	4/12	3/12	2/12	9/60
	Chicken	0/12	1/12	NA	NA	NA	1/24
	Sheep	2/12	NA	5/12	NA	2/12	9/36
	Total	2/36	1/24	9/24	3/12	4/24	19/120

1 Enrichment broths were subjected to direct plating from July to December 2008 or to immunomagnetic separation (IMS) from January to June 2009.

2 Sampling sites correspond to regions in the Culiacan Valley, Sinaloa, Mexico, as shown in Figure 1.

3 NA, None available.

In the subsequent sampling period (January to June 2009), the isolation method was modified for the recovery of STEC. A second set of 120 fecal samples were collected from farm animals at the same five sampling sites (Figure 1), and enrichment broths from the fecal samples were subjected to an immunomagnetic separation (IMS) method, followed by plating onto the indicator media. By using the IMS method, presumptive *E. coli* colonies were recovered from 15.8% (19/120) of the fecal samples tested (Table 1). Although all sampling sites had at least one enrichment broth that yielded presumptive colonies, 37.5% (9/24) of the positive samples were collected at farms in sampling site C, followed by sampling site D with 25.0% (3/12) of the positive samples. A lower number of positive samples were obtained from the other sampling sites (sites A, B, and E) (Table 1). Presumptive STEC colonies were mostly identified in 25.0% (9/36) of the enrichment broths from sheep feces, followed by 15.0% (9/60) from cattle feces and 4.2% (1/24) from chicken feces. From 25 enrichment broths that were positive by using both direct plating and IMS methods (Table 1), a total of 65 presumptive STEC isolates were isolated, and 83.1% (54/65) of these isolates were recovered from samples processed by the IMS method (Tables 2 and 3).

### Identification of Serotypes and Virulence Factors

The presumptive STEC isolates were examined by PCR for the presence of O-antigen and H-antigen genes that have been proposed to be associated with human disease. In addition, isolates were examined by PCR for the presence of the virulence genes *stx1, stx2, eae,* and *ehxA* (Tables 2 and 3). The results from these analyses showed that 43.1% (28/65) of all isolates were positive for the O157 O-antigen serogroup, and 40.0% (26/65) of all isolates were specifically serotype O157:H7 from 13 positive fecal samples that yielded at least one O157 isolate (Table 2). Interestingly, O157:H7 was a predominant serotype in the Culiacan Valley, being isolated from 3 of 5 sampling sites. *E. coli* O157:H7 isolates were recovered from chicken, cattle and sheep feces and were found to harbor the same virulence genes *stx2, eae* and *ehxA* (Table 2). Also identified were two *eae*-positive O157 isolates (O157:H4 and O157:NT) without *stx* genes, recovered from the same cattle fecal sample from sampling site E.

**Table 2:** List of *E. coli* O157 isolates used in this study.

Isolate	Fecal Sample	Serotype^1^	Sampling date^2^	Source	Sampling site^3^	Genes identified					
						*stx1*	*stx2*	*eae*		*ehxA*	
RM8744	Dc10	O157:H7	18-Nov-08	Cattle	D	–	+	+		+	
RM8753	Cs11	O157:H7	02-Dec-08	Sheep	C	–	+	+		+	
RM8754	Dc10	O157:H7	18-Nov-08	Cattle	D	–	+	+		+	
RM8759	Cs14	O157:H7	20-Jan-09	Sheep	C	–	+	+		+	
RM8767	Cc14	O157:H7	20-Jan-09	Cattle	C	–	+	+		+	
RM8768	Cc14	O157:H7	20-Jan-09	Cattle	C	–	+	+		+	
RM8769	Cc14	O157:H7	20-Jan-09	Cattle	C	–	+	+		+	
RM8771	Cc14	O157:H7	20-Jan-09	Cattle	C	–	+	+		+	
RM8781	Cs17	O157:H7	25-Feb-09	Sheep	C	–	+	+		+	
RM8920	Cs17	O157:H7	25-Feb-09	Sheep	C	–	+	+		+	
RM8921	Cs17	O157:H7	25-Feb-09	Sheep	C	–	+	+		+	
RM8922	Cc18	O157:H7	10-Mar-09	Cattle	C	–	+		+		+
RM8927	Ec18	O157:NT	10-Mar-09	Cattle	E	–	–		+		–
RM8928	Ec18	O157:H4	10-Mar-09	Cattle	E	–	–		+		–
RM9450	Cs18	O157:H7	10-Mar-09	Sheep	C	–	+		+		+
RM9451	Cs18	O157:H7	10-Mar-09	Sheep	C	–	+		+		+
RM9452	Cs23	O157:H7	26-May-09	Sheep	C	–	+		+		+
RM9453	Cs23	O157:H7	26-May-09	Sheep	C	–	+		+		+
RM9454	Cc23	O157:H7	26-May-09	Cattle	C	–	+		+		+
RM9455	Cc23	O157:H7	26-May-09	Cattle	C	–	+		+		+
RM9456	Cc20	O157:H7	07-Apr-09	Cattle	C	–	+		+		+
RM9457	Cc20	O157:H7	07-Apr-09	Cattle	C	–	+		+		+
RM9458	Bh19	O157:H7	24-Mar-09	Chicken	B	–	+		+		+
RM9459	Bh19	O157:H7	24-Mar-09	Chicken	B	–	+		+		+
RM9460	Cc20	O157:H7	07-Apr-09	Cattle	C	–	+		+		+
RM9461	Cc20	O157:H7	07-Apr-09	Cattle	C	–	+		+		+
RM9462	Dc24	O157:H7	03-Jun-09	Cattle	D	–	+		+		+
RM9463	Cs18	O157:H7	10-Mar-09	Sheep	C	–	+		+		+

1 NT, H-antigen non-typeable; ONT, O-antigen non-typeable.

2 Enrichment broths subjected to direct plating from July to December 2008 or to immunomagnetic separation from January to June 2009.

3 Sampling sites correspond to regions in the Culiacan Valley, Sinaloa, Mexico, as show in Figure 1.

Several important non-O157 serotypes, previously associated with a significant number of human infections [4], [19], were also isolated from 17 positive fecal samples that yielded at least one non-O157 isolate (Table 3). Two O111:H8 isolates were recovered from sheep feces at sampling site C and were positive by PCR for *stx1, eae* and *ehxA* genes (Table 3). Serotypes O8:NT and O8:H19, implicated in human illness but less frequently than serotype O111 [4], [19], were recovered from sheep feces at site C and from cattle feces at sites D and E, respectively. Furthermore, serotypes O146:H21 and O146:H8 together were identified in 7.7% (5/65) of the recovered isolates in the Culiacan Valley. All isolates with the O146 serogroup were isolated from sheep feces at sites A and E, corresponding to the two sampling locations that were furthest apart geographically (Table 3 and Figure 1). The most predominant non-O157 serotype, identified in 18.5% (12/65) of the recovered isolates, was O75:H8. Most of the O75:H8 isolates were recovered from sheep feces from site C and were positive by PCR for *stx1*, *stx2*, and *ehxA*, the same profile as O8 and O146 isolates (Table 3). Other identified serotypes, not associated previously with human illness [19], were O15:NT, O20:H4, O73:H4, O73:NT, O168:NT (Table 3).

**Table 3:** List of *E. coli* non-O157 isolates used in this study.

Isolate	FecalSample	Serotype^1^	Sampling date^2^	Source	Sampling site^3^	Genes identified					
						*stx1*	*stx2*	*eae*		*ehxA*	
RM8745	Es11	O73:H4	02-Dec-08	Sheep	E	+	+	–		+	
RM8746	Es11	O73:H4	02-Dec-08	Sheep	E	+	+	–		+	
RM8747	Bc2	O15:NT	22-Jul-08	Cattle	B	–	+	+		+	
RM8748	Bc2	O73:NT	22-Jul-08	Cattle	B	–	+	+		+	
RM8749	Es3	O20:H4	12-Aug-08	Sheep	E	–	+	–		–	
RM8750	Es3	O20:H4	12-Aug-08	Sheep	E	–	+	–		–	
RM8751	Es3	O20:H4	12-Aug-08	Sheep	E	–	+	–		–	
RM8752	Cs7	O75:H8	07-Oct-08	Sheep	C	+	+	–		+	
RM8755	Cs14	O111:H8	20-Jan-09	Sheep	C	+	–	+		+	
RM8756	As14	O146:H21	20-Jan-09	Sheep	A	+	+	–		+	
RM8757	As14	O146:H21	20-Jan-09	Sheep	A	+	+	–		+	
RM8758	As14	O146:H21	20-Jan-09	Sheep	A	+	+	–		+	
RM8760	Cs14	O75:H8	20-Jan-09	Sheep	C	+	+	–		+	
RM8761	Es14	O146:H21	20-Jan-09	Sheep	E	+	+	–		+	
RM8762	Es14	O146:H8	20-Jan-09	Sheep	E	+	+	–		+	
RM8763	Cs15	O75:H8	03-Feb-09	Sheep	C	+	+	–		+	
RM8764	Cs15	O75:H8	03-Feb-09	Sheep	C	+	+	–		+	
RM8765	Cs15	O75:H8	03-Feb-09	Sheep	C	+	+	–		+	
RM8766	Cs15	O8:NT	03-Feb-09	Sheep	C	–	+	–		+	
RM8770	As13	ONT:NT	08-Jan-09	Sheep	A	+	+	–		+	
RM8772	Ec16	O8:H19	17-Feb-09	Cattle	E	+	+	–		+	
RM8773	Ec16	O8:H19	17-Feb-09	Cattle	E	+	+	–		+	
RM8774	Ec16	O8:H19	17-Feb-09	Cattle	E	+	+	–		+	
RM8775	Ec16	O8:H19	17-Feb-09	Cattle	E	+	+	–		+	
RM8776	Dc15	O8:H19	03-Feb-09	Cattle	D	+	+	–		+	
RM8778	Cs17	O75:H8	25-Feb-09	Sheep	C	+	+	–		+	
RM8779	Cs17	O75:H8	25-Feb-09	Sheep	C	+	+	–		+	
RM8780	Cs17	O75:H8	25-Feb-09	Sheep	C	+	+	–		+	
RM8916	Cs14	O111:H8	20-Jan-09	Sheep	C	+	–	+		+	
RM8917	Dc14	O168:NT	20-Jan-09	Cattle	D	–	+	–		–	
RM8923	Cc18	O75:H8	10-Mar-09	Cattle	C	+	+		–		+
RM8924	Es18	ONT:H4	10-Mar-09	Sheep	E	+	+		–		+
RM8925	Es18	ONT:H4	10-Mar-09	Sheep	E	+	+		–		+
RM8926	Es18	ONT:H4	10-Mar-09	Sheep	E	+	+		–		+
RM8929	Cs18	O75:H8	10-Mar-09	Sheep	C	+	+		–		+
RM8930	Cs18	O75:H8	10-Mar-09	Sheep	C	+	+		–		+
RM13865	Cc20	O75:H8	07-Apr-09	Cattle	C	+	+		–		–

1 NT, H-antigen non-typeable; ONT, O-antigen non-typeable.

2 Enrichment broths subjected to direct plating from July to December 2008 or to immunomagnetic separation from January to June 2009.

3 Sampling sites correspond to regions in the Culiacan Valley, Sinaloa, Mexico, as show in Figure 1.

### Genotyping O157 STEC Isolates by PFGE and MLVA

**Figure 2:**
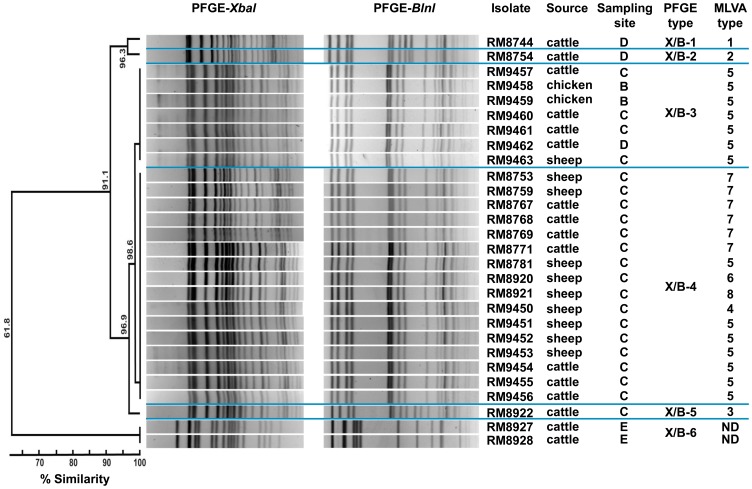
Analysis of the genetic relatedness among the O157 STEC isolates by PFGE and MLVA methods. Dendograms of the combined PFGE types with *Xba*I and *Bnl*I restriction enzymes were constructed using Bionumerics software v6.1 with the Dice coefficient and the UPGMA method. MLVA types were designated to each unique profile after analysis of variable tandem repeats in 11 genomic loci using Bionumerics software. ND, Not determined.

A PFGE analysis was conducted to identify the genetic relatedness in the recovered O157 isolates from the Culiacan Valley. The results indicated six unique PFGE types (Figure 2). Five of the six PFGE types were found in isolates recovered exclusively from one sampling site. The PFGE types X/B-1 and X/B-2 had a 96.3% similarity and were found in cattle isolates from sampling site D, and two closely-related types (X/B-3 and X/B-4) were identified in 82.1% (23/28) of the O157 isolates with >98% homology (Figure 2). In particular, the PFGE type X/B-4 was observed in 57.1% (16/28) of the O157 isolates from cattle and sheep feces from sampling site C alone. In contrast, isolates with the PFGE type X/B-3 were recovered from the three types of animal feces at sampling sites B, C, and D. The *stx*-negative O157 isolates RM8927 and RM8928 (PFGE type X/B-6) were recovered only from site E and showed the greatest divergence with only 61.8% similarity when compared to all other O157 isolates (Figure 2).

**Figure 3:**
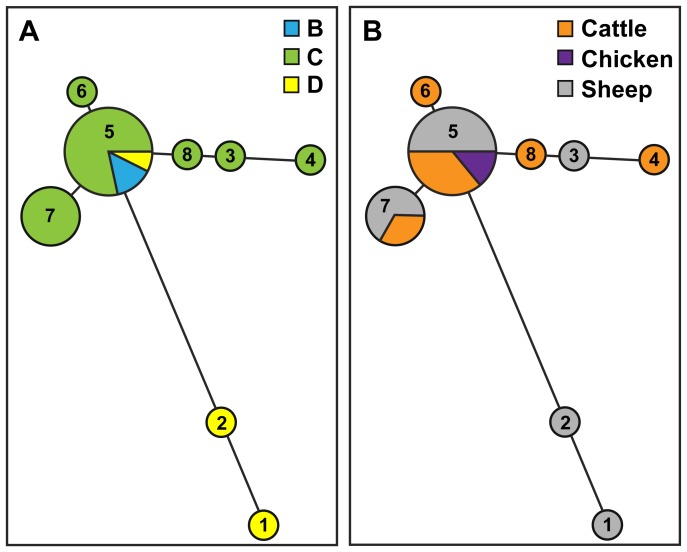
Phylogenetic relationships of MLVA types for the 26 O157:H7 STEC isolates. A minimum-spanning tree of MLVA types was generated with Bionumerics software v6.1 and the Manhattan distance algorithm. Each circle in the tree represents a different MLVA type, and the number in the circle indicates the MLVA type number. The circle size corresponds to the number of isolates with an identical MLVA type. Colors represent the sampling sites (A) or the sampling source (B) for each MLVA type.

As an alternate method for further differentiating the O157:H7 STEC isolates, MLVA was performed to identify polymorphisms in 11 variable number tandem repeat (VNTR) loci. The results indicated that the 11-loci MLVA method was able to discriminate among O157:H7 isolates that were indistinguishable by PFGE analysis (Figure 2). Among the 26 *E. coli* O157:H7 isolates that were examined, eight unique profiles were identified by MLVA as compared to only five PFGE types. Furthermore, MLVA type 5 (MLVA profile 35-10-12-15-7-6-13-5-8-2-4; Table S1) was the most common, representing 53.8% (14/26) of the O157:H7 isolates, and MLVA type 7 (MLVA profile 34-10-12-14-7-6-13-5-8-2-4; Table S1) was identified in 23% (6/26) of the O157:H7 isolates (Figure 2). To examine the relatedness among the identified MLVA types, a minimum spanning tree was constructed (Figure 3). The analysis revealed that five MLVA types were unique to isolates recovered from sampling site C (Figure 3A) but from various sources (Figure 3B). Only isolates with MLVA type 5 were identified at multiple sampling sites (Figure 3A). Furthermore, the distantly-related MLVA types 1 and 2 were identified in sheep isolates from site D.

The MLVA assay also revealed that the genetic diversity varied considerably at some VNTR loci in the O157:H7 isolates. For example, the number of tandem repeats at Vhec1 locus varied from 21 to 45 for isolates RM8744 and RM9450, respectively (Table S1). By contrast, the number of tandem repeats at Vhec5 locus was 7 for all isolates examined (Table S1). To further quantify the variation in the number of repeats at each VNTR locus among the O157 isolates, Nei’s diversity index was calculated (Table 4). As indicated by the diversity index, the degree of polymorphism ranged from 0 to 0.61 (Table 4). Vhec1 was the most variable locus with a total of 7 alleles. Vhec4 locus had intermediate diversity with 3 alleles, whereas no diversity was observed for Vhec5 with only one allele. The remaining eight VNTR loci that were included in this MLVA method were of low diversity for this set of O157:H7 isolates with diversity index values ranging from 0.07 to 0.21 (Table 4).

**Table 4:** Characterization of 11 variable-number tandem repeat loci in the O157:H7 STEC isolates used in this study.

	Variable Number Tandem Repeat loci										
	Vhec 1	Vhec 2	Vhec 3	Vhec 4	Vhec 5	Vhec 6	Vhec 7	O157-17	O157-19	O157-25	O157-35
Number of alleles	7	2	4	3	1	2	2	2	2	4	3
Tandem repeats range	21–45	9–10	8–12	14–17	7	6–9	7–13	3–5	7–8	1–5	4–8
Null alleles	No	No	No	No	No	No	No	No	No	No	No
Nei’s diversity index^1^	0.61	0.14	0.21	0.41	0	0.14	0.14	0.14	0.07	0.21	0.15

1 Nei’s diversity index for each locus was calculated as 1-∑(allele frequency)^2^ [67].

### Genotyping Non-O157 STEC Isolates by PFGE and MLVA

**Figure 4:**
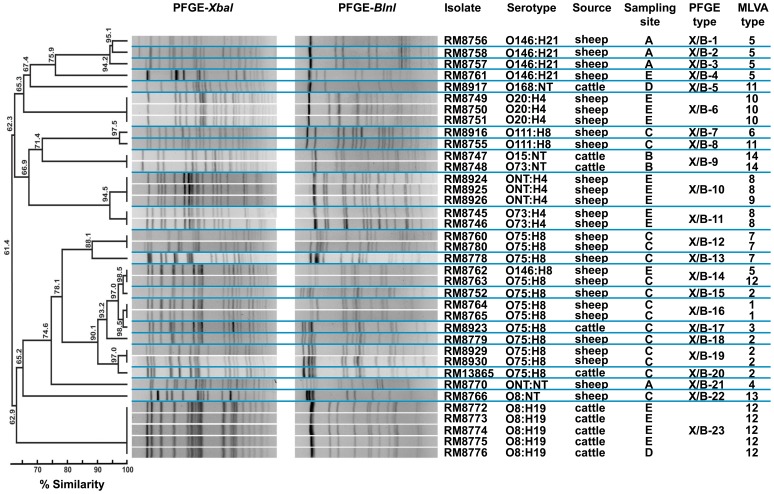
Analysis of the genetic relatedness among the non-O157 STEC isolates by PFGE and MLVA methods. Dendograms of the combined PFGE types with *Xba*I and *Bnl*I restriction enzymes were constructed using Bionumerics software v6.1 with the Dice coefficient and the UPGMA method. MLVA types were designated to each unique profile after analysis of tandem repeats in 10 genomic loci using Bionumerics software.

The PFGE analysis of 37 non-O157 STEC isolates revealed 23 distinct PFGE types with 61.4% similarity (Figure 4). Twenty-one of the PFGE types corresponded to non-O157 isolates that were recovered exclusively from only one of the sampling sites and from either cattle or sheep feces. Only PFGE types X/B-14 and X/B-23 were identified among non-O157 isolates from two different sampling sites (Figure 4). Some PFGE types were associated with a particular serotype. In particular, PFGE types X/B-6, X/B-11 and X/B-23 were only identified in isolates of serotypes O20:H4, O73:H4 and O8:H19, respectively, and predominantly from sampling site E. To further examine the genetic relatedness of the isolates, MLVA was performed by examining the polymorphisms in 10 genomic repeat-containing loci (Figure 4), targeting different loci than those used for the O157:H7 serotype (Table S2). The 10-loci MLVA identified 14 different MLVA types among the 37 non-O157 isolates analyzed and differentiated non-O157 isolates that could not be discriminated by PFGE (type X/B-14) (Figure 4). By contrast, this MLVA method was less discriminating than PFGE for distinguishing serotype O146:H21 isolates since the different PFGE types found in O146:H21 isolates were all MLVA type 5. MLVA type 10 was only identified in serotype O20:H4 isolates from sheep, and no other MLVA type was observed to be serotype specific (Figure 4). Multiple PFGE and MLVA types were identified in O75:H8 isolates, indicating that isolates with this serotype were the most diverse.

MLVA type 12 (MLVA profile 8-3-NA-8-3-10-1-15-NA-21; Table S2) was identified in 16.2% (6/37) of the non-O157 isolates. The other common MLVA types, MLVA type 2 (MLVA profile 6-3-NA-7-3-7-1-2-NA-15) and MLVA type 5 (MLVA profile 6-3-NA-8-3-4-1-2-NA-15), were each detected in 13.5% (5/37) of the non-O157isolates (Table S2). The number of alleles detected per locus ranged from 1 (locus CVN015) to 9 (locus CVN014) (Table 5). In the set of non-O157 isolates analyzed in this study, five loci (CVN002, CVN003, CVN016, CVN017 and CCR001) had null alleles (Tables 5 and S2). Interestingly, locus CVN003 could only be amplified in serotypes that were positive for the flagellin H4 antigen (O20:H4, O73:H4 and ONT:H4) (Table S2), suggesting either sequence polymorphisms at priming sites in serotypes that had null alleles or the absence of this locus. Further analysis of the allelic polymorphisms by calculating Nei’s diversity index revealed that three loci (CVN004, CVN014 and CVN016) were among the most diverse (Table 5). In particular, locus CVN014 was the most polymorphic locus, as has been observed in other studies [43]–[45]. The diversity index calculation also indicated that two loci had intermediate diversity (CVN001 and CCR001), whereas five loci resulted in low or no diversity (CVN002, CVN003, CVN007, CVN015 and CVN017) (Table 5).

**Table 5:** Characterization of 10 genomic-repeat containing loci in the non-O157 STEC isolates used in this study.

	Genomic repeat-containing loci									
	CVN001	CVN002	CVN003	CVN004	CVN007	CVN014	CVN015	CVN016	CVN017	CCR001
Number of alleles	3	3	3	6	2	9	1	6	2	3
Tandem repeats range	5–8	1–3	2–5	4–13	3–4	4–15	1	1–15	9	15–21
Null alleles	No	Yes	Yes	No	No	No	No	Yes	Yes	Yes
Nei’s diversity index^1^	0.43	0.20	0.36	0.74	0.10	0.83	0	0.70	0.10	0.51

1 Nei’s diversity index for each locus was calculated as 1−∑(allele frequency)^2^ [67].

**Figure 5:**
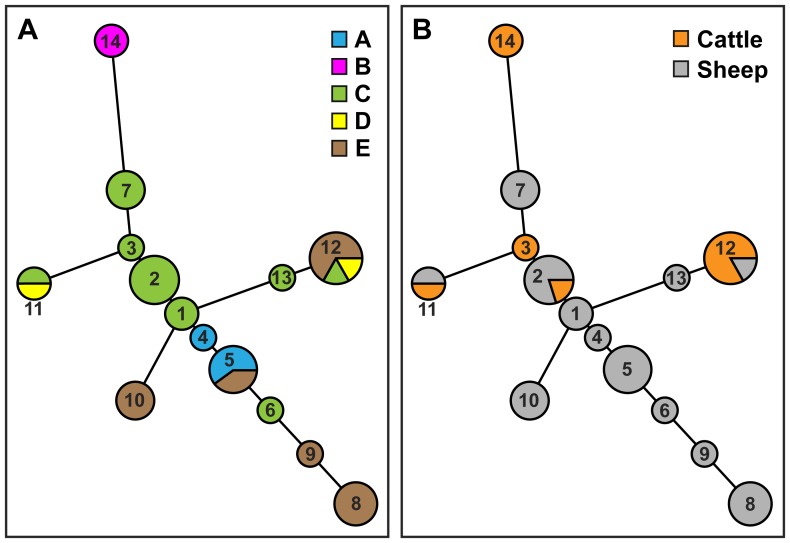
Phylogenetic relationships of MLVA types for the 37 non-O157 STEC isolates. A minimum-spanning tree was generated with Bionumerics software v61 and the Manhattan distance algorithm. Each circle in the tree represents a different MLVA type, and the number in the circle indicates the MLVA type number. The circle size corresponds to the number of isolates with an identical MLVA type. Colors represent the sampling sites (A) or the sampling source (B) for each MLVA type.

A minimum spanning tree of MLVA types indicated that MLVA type 14 was more distantly-related when compared to the other MLVA types (Figure 5). Furthermore, MLVA type 14 was only identified in isolates recovered from sampling site B (Figure 5A) and only from cattle feces (Figure 5B). The minimum spanning tree also revealed that sampling site C was a source of MLVA types 1, 2, 3, and 7 that were closely related (Figure 5A). Interestingly, sheep were a primary source of non-O157 isolates with diverse MLVA types (Figure 5B).

## Discussion

The present study investigated the prevalence of zoonotic STEC in small farms within rural communities, located in the agricultural Culiacan Valley along the coastal northwestern part of Mexico. The Culiacan Valley is considered to be one of the most important agricultural regions in Mexico [41]. The farms, examined in this study, were located in rural communities where livestock was often raised without an efficient management of animal wastes [42]. Thus, a lack of good agricultural practices could potentially contribute to the dispersal and transmission of pathogens throughout the environment. An understanding of the primary animal reservoirs in rural communities within an agricultural region would be imperative for the development of control measures of any risk factors that could lead to human infections with zoonotic STEC.

Only a limited number of studies have previously documented the prevalence of STEC in animal reservoirs in various locations in Mexico. The contamination of animal carcasses with O157:H7 and non-O157 STEC was detected in slaughter plants sampled in northeast and western Mexico [38], [39]. The recovered STEC isolates from these slaughter plants were found positive for the virulence factors intimin and hemolysin. A separate study confirmed the presence of *E. coli* O157:H7 in animal feces collected from dairy and beef cattle farms and from swine-farrowing facilities throughout Central Mexico [36]. Whether the recovered O157 isolates from the farms in Central Mexico harbor genes associated with severe human illness is still to be determined. Thus, additional studies are needed to identify the prevalence and phylogenetic relatedness of O157 and non-O157 STEC in other relevant agricultural regions of Mexico.

Given that cattle and other ruminants are considered relevant reservoirs of zoonotic STEC [1], [18], [19], [21], the present study is the first report that has examined the prevalence of O157 and non-O157 STEC from feces of domestic animals that were raised on the sampled rural farms in the agricultural Culiacan Valley. The results from this study indicated that O157 STEC were detected in 5.4% (13/240) and non-O157 STEC were detected in 7.1% (17/240) of the animal fecal samples. The observed STEC prevalence in farm animals in Mexico’s Culiacan Valley appears to be lower when compared to some countries. Moreover, a published report that comprehensively examined the worldwide prevalence of O157 and non-O157 in livestock feces reported significant wide ranges [46]. In particular, the prevalence of O157 ranged from 0.2% to 27.8% and of non-O157 ranged from 2.1% to 70.1% [46]. These observations have also indicated that multiple factors, including sampling locations, hosts, periods of study and methods, may contribute to the dynamic nature observed among studies of pathogen prevalence in animals [18], [35], [46], [47].

Due to the complexity of detecting and enumerating toxigenic *E. coli* in naturally-contaminated samples [18], [48], an enrichment method and plating on selective media was employed to increase the efficiency for isolating both O157 and non-O157 STEC. The use of the IMS method facilitated significantly higher recovery of isolates when compared to direct plating. The number of fecal samples that were positive for presumptive STEC using the IMS method was 2.1-times higher than those subjected to direct plating on selective media. These findings are in agreement with other studies that have reported an increased effectiveness in STEC detection in animal feces by using the IMS method [23], [49]–[51]. Furthermore, the increased recovery of STEC isolates that was observed during the second sampling period can be attributed to performing the enrichment step without antibiotics, a procedure proposed to increase the number of STEC cells before exposure to the selective conditions in the isolation media [52].

The O157:H7 serotype was identified in 40% (26/65) of the *E. coli* isolates that were recovered from the domestic animals sampled in this study. Most O157 isolates were recovered from cattle feces and sheep feces. Interestingly, two O157:H7 isolates were recovered from chicken feces, considered to be a rare animal source for this serotype [18]. Genotyping studies revealed that most O157:H7 isolates grouped into two PFGE 2-enzyme profiles with 98.6% homology, thus, the use of MLVA as a genotyping tool facilitated further discrimination of isolates that were indistinguishable by PFGE. Further analysis of the allelic polymorphisms of the 11 VNTR loci of the O157 MLVA system indicated that most alleles had low or intermediate diversity in the O157:H7 isolates from the Culiacan Valley. Vhec1 was the only VNTR locus that was identified to have high allelic diversity. This finding is in agreement with previous reports that documented a particularly high polymorphism at Vhec1 [53]–[55]. The limited allelic diversity in conjunction with the observation that all O157:H7 isolates had the same virulence profile (positive for *stx2, eae, ehxA*) suggests that the O157:H7 isolates from this region in Mexico are highly related. It also appears that a small number of genetic types may predominate and persist in different animal reservoirs on the rural farms. In particular, the O157:H7 sheep isolate RM8753 was recovered by direct plating, and six months later, genotypically-identical isolates by MLVA and PFGE were recovered by the IMS method from the same sampling site (site C), but from both sheep and cattle. This finding may suggest persistence of the O157:H7 isolates in the Culiacan Valley and a potential transmission between species.

The non-O157 serotypes O8:H19, O75:H8, O111:H8 and O146:H21 were identified in 35.4% (23/65) of the STEC isolates that were recovered in the Culiacan Valley. These relevant serotypes have been previously implicated with disease outbreaks or with severe symptoms in humans [3], [4], [19]. The non-O157 isolates were predominantly recovered from sheep and were identified to harbor either one or both *stx* genes. With the exception of five isolates, all other non-O157 isolates (86.5%, 32/37) were identified to be positive for *ehxA.* However, only 10.8% (4/37) of the non-O157 isolates harbored *eae* gene. Similar to what was observed in the analysis of O157:H7 isolates, a diverse set of non-O157 isolates was identified at sampling site C, located in a valley between two large urban cities (Culiacan and Navolato) and proximal to the mountain ranges along the east.

Among the non-O157 serotypes that were identified in this study, serotype O75:H8 was the most variable serotype by both PFGE and MLVA. The O75:H8 isolates, representing 18.5% (12/65) of the isolates recovered from the Culiacan Valley, were a common sheep serotype. Another sheep serotype was O146:H21. The predominant isolation of O75:H8 and O146:H21 isolates from sheep was in agreement with previous reports that documented the same animal source for these non-O157 serotypes in several countries such as Australia, Brazil, New Zealand, and Spain [16], [29], [30], [56], [57]. Moreover, to our knowledge, the present study is the first report of sheep feces as an animal source of STEC O111:H8, a serotype previously identified to be recovered exclusively from cattle [4], [56], [58], [59].

Genotypic analysis by PFGE and MLVA revealed that cattle isolates with serotype O8:H19 from the Culiacan Valley were closely related. By contrast, a high genotypic diversity was identified in O8:H19 isolates from beef products in Argentina [44]. In the present study, the low genomic diversity that was observed in the O8:H19 isolates may indicate that a prevalent genotype may be circulating in this geographical region in Mexico. Additional surveys would be needed to determine whether other STEC serotypes may also represent stable and predominant genotypes in this agricultural region in Mexico. Future work will investigate other sources of O157 and non-O157 STEC by examining wildlife and environmental samples (plants, soil, sediment, and irrigation water) to obtain a better understanding of pathogen diversity, persistence, and movement. Further assessment of additional molecular risk factors associated with STEC pathogenesis would also provide relevant information regarding the virulence potential of toxigenic *E. coli* recovered from the Culiacan Valley in Mexico.

## Materials and Methods

### Ethics Statement

No specific permits were obtained for the described field studies. Access to privately-owned land was provided after verbal agreement with land owners. The field studies did not involve endangered or protected species. No animals were harmed in the acquisition of fecal samples.

### Sample Collection

From July 2008 to June 2009, a total of 240 fecal samples were collected from different sampling sites in the Culiacan Valley in Northwest Mexico. The five sampling sites, Jotagua (A), Agua Caliente (B), Cofradia de Navolato (C), Iraguato (D), and El Castillo (E), were selected to be 14–38 km apart and to represent the study area in the Culiacan Valley (Figure 1). Sites A and B were located in the mountain hillsides with a semi-humid climate. Sites C and D were in close proximity to the urban areas of Navolato and Culiacan municipalities, and site E was located nearest to the coast (Figure 1). Sites C, D, and E had a semi-arid climate. Each sampling site was visited every two weeks and consisted of small farms of about 20 to 50 farm animals that were located in rural communities nearby rivers used for irrigation, where livestock raising is a common activity among local families. A fecal sample was collected from each species of animal present at each sampling site. Approximately, 100 g of fecal samples from asymptomatic cattle and sheep and 30 g of fecal samples from chicken were collected from different areas within each sampling site by using a sterile spatula and gloves, and placed into labeled sterile plastic bags, as in previous studies [42]. All collected fecal samples were transported immediately under refrigeration to the Food and Environmental Microbiology Laboratory (CIAD, Culiacan, Mexico) and then processed within the next 6 h.

### Isolation Method for STEC from Animal Fecal Samples

Fecal samples were collected during two sequential periods and were analyzed by separate isolation methods. From July to December 2008, STEC were isolated from 120 fecal samples by employing a direct plating method. A total of 25 g of animal feces were removed with sterile spatula and placing it in a sterile plastic bag. The sample was homogenized manually for 5 to 10 min, followed by addition of 225 ml of tryptic soy broth (TSB) (BD Bioxon, Nuevo Leon, Mexico), supplemented with 1.5 g/l bile salts (Sigma-Aldrich Chemie GmbH, Munich, Germany), 1.5 g/l dipotassium phosphate (Sigma-Aldrich), 0.0125 mg/l cefixime (Sigma-Aldrich), 10 mg/l cefsulodin sodium salt hydrate (Sigma-Aldrich), and 8 mg/l vancomycin hydrochloride (Sigma-Aldrich). The enrichment culture was incubated at 37°C for 18–24 h with constant shaking. A 50 µl aliquot of the enrichment cultures were spread on sorbitol MacConkey agar (CT-SMAC) (Difco, Detroit, MI, USA), containing 0.5 mg/l cefixime (Sigma-Aldrich) and 2.5 mg/l potassium tellurite hydrate (Sigma-Aldrich) [34] as well as on CHROMagar™ O157 (CHROMagar, Paris, France). Agar plates were then incubated for 18–24 h at 37°C. Presumptive colonies were selected based on purple colony color on CHROMagar or either a colorless or light gray color on CT-SMAC.

A total of 120 fecal samples were collected from January to June 2009 and were processed by including an IMS method, modified from a method described previously for isolation of O157 STEC from environmental samples [34]. Briefly, a total of 10 g of animal feces were homogenized as described above and were then added to 90 ml of TSB without antibiotics, as in previous studies [34]. The enrichment broths were incubated for 2 h at 25°C, then at 42°C for 8 h with constant shaking, and were held at 4°C until the following morning without shaking. A 500 µl sample of the enrichment culture and 500 µl of 1× phosphate buffer solution (PBS) were incubated with 10 µl of Dynabeads® anti-*E. coli* O157 magnetic beads (Life Technologies, Grand Island, NY, USA) for 30 min with constant mixing. The magnetic beads were then washed twice with PBS containing 0.05% Tween-20 by using a Dynal® BeadRetriever Tube Rack (Life Technologies) and finally resuspended in 100 µl of PBS. A 50 µl sample was plated on CHROMagar O157 and on Rainbow® Agar O157 (Biolog, Hayward, CA, USA). Presumptive colonies were selected based on purple color CHROMagar O157 and on the several colors for typical colonies on Rainbow Agar O157, as in previous studies [60].

### Identification of Serotypes and Virulence Gene Profiles

To determine whether the isolates, grown on selective indicator media, were authentic *E. coli* O157, a colony immunoblot assay was performed. Presumptive colonies and positive control O157 STEC strain RM1484 were patched on plates of Difco™ Luria Bertani (LB) agar (Fisher Scientific, Pittsburg, PA, USA) by using a numbered grid and grown at 37°C for 18 h and were then blotted onto Whatman Protran® BA85 nitrocellulose membrane filter discs (0.45 µm pore size, VWR International, Radnor, PA, USA). The nitrocellulose membranes were washed, blocked, incubated with anti-O157 IgG monoclonal antibody 13B3, followed by incubation with alkaline phosphatase-conjugated goat anti-mouse IgG antibody, following previously described procedures [34]. Positive signals on the membranes were detected after incubation with SIGMA*FAST*™ BCIP®/NBT colorimetric substrates (Sigma-Aldrich) [34].

Positive colonies with the anti-O157 antibody were then examined by real-time polymerase chain reaction (RT-PCR) after amplification of a 497-bp region of the *rfbE* gene, encoding the O157 O-antigen transporter, as described elsewhere [34]. As template for the RT-PCR reaction, bacteria were transferred from the patch plate directly into the PCR tube by using a sterile toothpick. The RT-PCR reaction was performed in a Stratagene MX3000P Real-Time PCR machine (Agilent Technologies, Santa Clara CA, USA) with the following conditions: 95°C for 5 min, and 60 cycles of 95°C for 15 sec and 60°C for 45 sec. Samples were considered positive for *rfbE* gene when the cycle threshold value of the RT-PCR assay was below 20, as in previous studies [34].

Presumptive colonies on selective media with atypical colony colors for O157 STEC were analyzed further by PCR to detect the *wzx* and *wzy* genes in the O-antigen gene cluster of 10 non-O157 serogroups (O26, O45, O91, O103, O104, O111, O113, O121, O128, and O145) using PCR primers published previously [61]. All isolates were screened for the presence of the flagellin *fliC*_H7_ gene, and the virulence genes encoding Shiga toxins (*stx1, stx2),* intimin *(eae),* and enterohemolysin *(exhA*) [61]. Detection of *fliC* genes, targeting additional H-types (H2, H8, H1, H19, H21, and H28), was performed as described previously [62]. Detection of flagellin H4 antigen was performed with primers 5′-GATAACCAGACGATCAGCATTGG-3′ (forward) and 5′-CTTCCGCTGCACCAACAGT-3′ (reverse). PCR reagents were supplied by Promega Corporation (Madison, WI, USA), and PCR primers were purchased from Eurofins MWG Operon (Huntsville, AL, USA). As template for the PCR reaction, bacterial cultures of the isolates were grown aerobically in TSB broth (Difco) for 24 h with constant shaking (200 rpm) at 37°C, and 100 µl of the bacterial cultures were collected by centrifugation at 2000×*g* for 5 min. Cell pellets were resuspended in 100 µl of HyPure™ molecular biology-grade water (HyClone Laboratories, Inc., Logan, UT, USA) and incubated at 95°C for 20 min, as in previous studies [63]. The lysates were centrifuged at 2000×*g* for 5 min, and the supernatants were collected and frozen until further use. PCR amplifications were performed in a 25 µl reaction mixture, each containing 5 µl of the bacterial crude lysate, 0.5µM of each primer and 12.5 µl of 2× GoTaq® Green Master Mix (Promega Corporation). The reaction mixtures were placed in a Dyad Peltier Thermal Cycler (Bio-Rad Laboratories, Hercules, CA, USA) with the following settings: 5 min at 94°C, followed by 25 cycles of 45 sec at 94°C, 1 min 60°C, 1 min at 72°C, and a final extension time of 7 min at 72°C. Amplified products were analyzed in 2% agarose gels containing 0.04 µl/ml GelRed Nucleic Acid Stain (Phenix Research, Candler, NC, USA). When the PCR assays for O- antigen were inconclusive, bacterial isolates were sent to the *E. coli* Reference Center at Pennsylvania State University, University Park, PA, USA.

### PFGE Molecular Subtyping

STEC isolates were typed with the standardized rapid PFGE protocol used by PulseNet laboratories, according to previous reports [64]. Isolates were grown for 14–18 h on LB agar and were resuspended in a cell suspension buffer (100 mM Tris, 100 mM EDTA, pH 8.0) to an OD_600_ 0.8–1.0, and an agarose plug was prepared by mixing 400 µl of the cell suspension with 400 µl of 1% SeaKem® Gold agarose (Lonza, Rockland, ME, USA) and 20 µl of proteinase K (Roche Diagnostics, Indianapolis, IN, USA) [64]. Agarose-embedded cells were lysed, and intact genomic DNA was digested with either *Xba*I or *Bln*I restriction enzymes (Roche Diagnostics) for 4 h at 37°C. The fragments were separated with a CHEF Mapper™ Pulsed-Field Electrophoresis System (BioRad, Hercules, CA, USA) for 20.5 h with switch times at 6 V/cm ranging from 2.16 to 54.17 s. The PulseNet universal standard strain, *Salmonella enterica* Braenderup H9812, was used as a molecular reference marker [64]. Gels were stained with ethidium bromide and were visualized under UV trans-illumination (Alpha Innotech Corp., San Leandro, CA, USA). PFGE profiles of the isolates with both digests were compared by performing band matching, and dendograms were constructed using Bionumerics software version 6.1 (Applied Maths, Austin, TX, USA) with the Dice coefficient and the unweighted pair-group method with arithmetic mean (UPGMA).

### MLVA Molecular Subtyping

MLVA typing of O157 STEC isolates was performed by using capillary electrophoresis after amplification of 11 VNTR loci in three multiplex PCR reactions [34], [53], [65]. Multiplex PCR forward primers were 5′-fluorescently labeled with 6FAM, NED, and VIC dyes [34], and all primers were purchased from Applied Biosystems-Life Technologies (Carlsbad, CA, USA). PCR reaction 1 amplified VNTR loci Vhec1, Vhec3, Vhec4, and Vhec5, and reaction 2 amplified VNTR loci Vhec1, Vhec2, Vhec6, and Vhec7, as in previous studies [34], [65]. VNTR loci O157–17, O157–19, O157–25, and O157–37 were amplified in reaction 3 [53]. PCR reaction mix and thermal cycling parameters were performed as described in a previous study [34]. For performing the MLVA typing in non-O157 STEC, ten genomic-repeat containing loci were amplified in four multiplex PCR reactions with fluorescent dye-conjugated primers [45], [66]. VNTR loci CVN003 and CVN014 were amplified in reaction 1, and loci CVN001, CVN004, CVN007 and CVN015 were amplified in reaction 2, as described previously [45]. PCR reaction 3 only amplified CVN002 locus [45], and loci CVN016, CVN017 and CCR001 were amplified in reaction 4 [66]. PCR reaction mix and thermal cycling parameters were performed as described in a previous study [45], [66].

After the PCR amplifications, the multiplex reactions for O157 STEC were pooled and diluted 1∶50 into distilled water. For non-O157 STEC, the multiplex PCR reactions were pooled as follows: 10 µl of reaction 1, 1.75 µl of reaction 2 and 5 µl of reaction 3. One µl of the pooled PCR reactions was added to 12 µl HiDi formamide (Applied Biosystems-Life Technologies) and 0.08 µl of ROX-labeled MapMarker 1000 size standard (Bioventures, Inc., Murfreesboro, TN). For reaction 4, 1 µl of a 1∶50 dilution was added to 10 µL HiDi formamide (Applied Biosystems-Life Technologies) and 0.04 µL of ROX-labeled MapMarker 1000 size standard (Bioventures, Inc.). The mixtures were size-fractionated with the Applied Biosystems 3130*xl* Genetic Analyzer, and the fragments sizes were determined with the GeneMapper® software v3.7 (Applied Biosystems-Life Technologies) and were then converted to number of tandem repeats [34], [53]. Each new multiple of tandem repeat was assigned to a distinct allele number, and the allele numbers per locus were determined using BioNumerics software v6.1 to designate MLVA types to each unique MLVA profile. For data analysis, an arbitrary number (−2) was assigned to null alleles, where no amplification product was detected [53]. Cluster analysis and minimal spanning trees of MLVA types were constructed using BioNumerics software v6.1 and the Manhattan distance algorithm to determine the genetic relationships among the isolates. The Nei’s marker diversity index, ranging from 0 (no diversity) to 1 (complete diversity), was calculated for each locus by using the equation 1−∑(allele frequency)^2^ [67], as in previous studies [55], [68]. Null alleles were included in the diversity index calculation.

## Supporting Information

Table S1 **MLVA profiles for each unique MLVA type identified in the STEC O157:H7 isolates used in this study.**
(DOCX)

Table S2 **MLVA profiles for each unique MLVA type identified in the STEC non-O157 isolates used in this study.**
(DOCX)
