# Supplementary material for: Genotypic Analyses of Shiga Toxin-Producing Escherichia coli O157 and Non-O157 Recovered from Feces of Domestic Animals on Rural Farms in Mexico
Source: PLoS One. 2012 Dec 10;7(12):e51565. doi: 10.1371/journal.pone.0051565 (PMC3519732; doi:10.1371/journal.pone.0051565)
Supplement: Table S1 — MLVA profiles for each unique MLVA type identified in the STEC O157:H7 isolates used in this study. (DOCX) [file pone.0051565.s001.docx]

**Table S1.** MLVA profiles for each unique MLVA type identified in the STEC O157:H7 isolates used in this study.

| **Isolate** | **MLVA type** | **Number of tandem repeats in designated locus** | | | | | | | | | | |
| --- | --- | --- | --- | --- | --- | --- | --- | --- | --- | --- | --- | --- |
|  |  | **Vhec 1** | **Vhec 2** | **Vhec 3** | **Vhec 4** | **Vhec 5** | **Vhec 6** | **Vhec 7** | **O157-17** | **O157-19** | **O157-25** | **O157-35** |
| RM8744 | 1 | 21 | 9 | 8 | 17 | 7 | 9 | 7 | 3 | 7 | 4 | 6 |
| RM8754 | 2 | 25 | 9 | 9 | 15 | 7 | 9 | 7 | 3 | 8 | 5 | 8 |
| RM8922 | 3 | 38 | 10 | 12 | 15 | 7 | 6 | 13 | 5 | 8 | 2 | 4 |
| RM9450 | 4 | 45 | 10 | 12 | 15 | 7 | 6 | 13 | 5 | 8 | 2 | 4 |
| RM8781 | 5 | 35 | 10 | 12 | 15 | 7 | 6 | 13 | 5 | 8 | 2 | 4 |
| RM9451 | 5 | 35 | 10 | 12 | 15 | 7 | 6 | 13 | 5 | 8 | 2 | 4 |
| RM9452 | 5 | 35 | 10 | 12 | 15 | 7 | 6 | 13 | 5 | 8 | 2 | 4 |
| RM9453 | 5 | 35 | 10 | 12 | 15 | 7 | 6 | 13 | 5 | 8 | 2 | 4 |
| RM9454 | 5 | 35 | 10 | 12 | 15 | 7 | 6 | 13 | 5 | 8 | 2 | 4 |
| RM9455 | 5 | 35 | 10 | 12 | 15 | 7 | 6 | 13 | 5 | 8 | 2 | 4 |
| RM9456 | 5 | 35 | 10 | 12 | 15 | 7 | 6 | 13 | 5 | 8 | 2 | 4 |
| RM9457 | 5 | 35 | 10 | 12 | 15 | 7 | 6 | 13 | 5 | 8 | 2 | 4 |
| RM9458 | 5 | 35 | 10 | 12 | 15 | 7 | 6 | 13 | 5 | 8 | 2 | 4 |
| RM9459 | 5 | 35 | 10 | 12 | 15 | 7 | 6 | 13 | 5 | 8 | 2 | 4 |
| RM9460 | 5 | 35 | 10 | 12 | 15 | 7 | 6 | 13 | 5 | 8 | 2 | 4 |
| RM9461 | 5 | 35 | 10 | 12 | 15 | 7 | 6 | 13 | 5 | 8 | 2 | 4 |
| RM9462 | 5 | 35 | 10 | 12 | 15 | 7 | 6 | 13 | 5 | 8 | 2 | 4 |
| RM9463 | 5 | 35 | 10 | 12 | 15 | 7 | 6 | 13 | 5 | 8 | 2 | 4 |
| RM8920 | 6 | 35 | 10 | 12 | 15 | 7 | 6 | 13 | 5 | 8 | 1 | 4 |
| RM8753 | 7 | 34 | 10 | 12 | 14 | 7 | 6 | 13 | 5 | 8 | 2 | 4 |
| RM8759 | 7 | 34 | 10 | 12 | 14 | 7 | 6 | 13 | 5 | 8 | 2 | 4 |
| RM8767 | 7 | 34 | 10 | 12 | 14 | 7 | 6 | 13 | 5 | 8 | 2 | 4 |
| RM8768 | 7 | 34 | 10 | 12 | 14 | 7 | 6 | 13 | 5 | 8 | 2 | 4 |
| RM8769 | 7 | 34 | 10 | 12 | 14 | 7 | 6 | 13 | 5 | 8 | 2 | 4 |
| RM8771 | 7 | 34 | 10 | 12 | 14 | 7 | 6 | 13 | 5 | 8 | 2 | 4 |
| RM8921 | 8 | 37 | 10 | 11 | 15 | 7 | 6 | 13 | 5 | 8 | 2 | 4 |
