# Supplementary material for: Genotypic Analyses of Shiga Toxin-Producing Escherichia coli O157 and Non-O157 Recovered from Feces of Domestic Animals on Rural Farms in Mexico
Source: PLoS One. 2012 Dec 10;7(12):e51565. doi: 10.1371/journal.pone.0051565 (PMC3519732; doi:10.1371/journal.pone.0051565)
Supplement: Table S2 — MLVA profiles for each unique MLVA type identified in the STEC non-O157 isolates used in this study. (DOCX) [file pone.0051565.s002.docx]

**Table S2.** MLVA profiles for each unique MLVA type identified in the STEC non-O157 isolates used in this study.

| **Isolate** | **Serotype^1^** | **MLVA type** | **Number of repeats in designated locus^2^** | | | | | | | | | |
| --- | --- | --- | --- | --- | --- | --- | --- | --- | --- | --- | --- | --- |
|  |  |  | **CVN001** | **CVN002** | **CVN003** | **CVN004** | **CVN007** | **CVN014** | **CVN015** | **CVN016** | **CVN017** | **CCR001** |
| RM8764 | O75:H8 | 1 | 6 | 3 | NA | 7 | 3 | 6 | 1 | 2 | NA | 15 |
| RM8765 | O75:H8 | 1 | 6 | 3 | NA | 7 | 3 | 6 | 1 | 2 | NA | 15 |
| RM8752 | O75:H8 | 2 | 6 | 3 | NA | 7 | 3 | 7 | 1 | 2 | NA | 15 |
| RM8779 | O75:H8 | 2 | 6 | 3 | NA | 7 | 3 | 7 | 1 | 2 | NA | 15 |
| RM8929 | O75:H8 | 2 | 6 | 3 | NA | 7 | 3 | 7 | 1 | 2 | NA | 15 |
| RM8930 | O75:H8 | 2 | 6 | 3 | NA | 7 | 3 | 7 | 1 | 2 | NA | 15 |
| RM13865 | O75:H8 | 2 | 6 | 3 | NA | 7 | 3 | 7 | 1 | 2 | NA | 15 |
| RM8923 | O75:H8 | 3 | 6 | 3 | NA | 7 | 3 | 8 | 1 | 2 | NA | 15 |
| RM8770 | ONT:NT | 4 | 6 | 3 | NA | 7 | 3 | 5 | 1 | 2 | NA | 15 |
| RM8756 | O146:H21 | 5 | 6 | 3 | NA | 8 | 3 | 4 | 1 | 2 | NA | 15 |
| RM8757 | O146:H21 | 5 | 6 | 3 | NA | 8 | 3 | 4 | 1 | 2 | NA | 15 |
| RM8758 | O146:H21 | 5 | 6 | 3 | NA | 8 | 3 | 4 | 1 | 2 | NA | 15 |
| RM8761 | O146:H21 | 5 | 6 | 3 | NA | 8 | 3 | 4 | 1 | 2 | NA | 15 |
| RM8762 | O146:H8 | 5 | 6 | 3 | NA | 8 | 3 | 4 | 1 | 2 | NA | 15 |
| RM8916 | O111:H8 | 6 | 6 | NA | NA | 8 | 3 | 4 | 1 | 2 | NA | 15 |
| RM8760 | O75:H8 | 7 | 6 | 3 | NA | 7 | 3 | 15 | 1 | 2 | NA | 15 |
| RM8778 | O75:H8 | 7 | 6 | 3 | NA | 7 | 3 | 15 | 1 | 2 | NA | 15 |
| RM8780 | O75:H8 | 7 | 6 | 3 | NA | 7 | 3 | 15 | 1 | 2 | NA | 15 |
| RM8745 | O73:H4 | 8 | 6 | 3 | 2 | 10 | 3 | 4 | 1 | 1 | NA | 21 |
| RM8746 | O73:H4 | 8 | 6 | 3 | 2 | 10 | 3 | 4 | 1 | 1 | NA | 21 |
| RM8924 | ONT:H4 | 8 | 6 | 3 | 2 | 10 | 3 | 4 | 1 | 1 | NA | 21 |
| RM8925 | ONT:H4 | 8 | 6 | 3 | 2 | 10 | 3 | 4 | 1 | 1 | NA | 21 |
| RM8926 | ONT:H4 | 9 | 6 | NA | NA | 10 | 3 | 4 | 1 | 1 | NA | 21 |
| RM8749 | O20:H4 | 10 | 5 | 1 | 5 | 4 | 3 | 6 | 1 | NA | NA | 15 |
| RM8750 | O20:H4 | 10 | 5 | 1 | 5 | 4 | 3 | 6 | 1 | NA | NA | 15 |
| RM8751 | O20:H4 | 10 | 5 | 1 | 5 | 4 | 3 | 6 | 1 | NA | NA | 15 |
| RM8755 | O111:H8 | 11 | 6 | 3 | NA | 5 | 3 | 9 | 1 | 8 | 9 | 15 |
| RM8917 | O168:NT | 11 | 6 | 3 | NA | 5 | 3 | 9 | 1 | 8 | 9 | 15 |
| RM8763 | O75:H8 | 12 | 8 | 3 | NA | 8 | 3 | 10 | 1 | 15 | NA | 21 |
| RM8772 | O8:H19 | 12 | 8 | 3 | NA | 8 | 3 | 10 | 1 | 15 | NA | 21 |
| RM8773 | O8:H19 | 12 | 8 | 3 | NA | 8 | 3 | 10 | 1 | 15 | NA | 21 |
| RM8774 | O8:H19 | 12 | 8 | 3 | NA | 8 | 3 | 10 | 1 | 15 | NA | 21 |
| RM8775 | O8:H19 | 12 | 8 | 3 | NA | 8 | 3 | 10 | 1 | 15 | NA | 21 |
| RM8776 | O8:H19 | 12 | 8 | 3 | NA | 8 | 3 | 10 | 1 | 15 | NA | 21 |
| RM8766 | O8:NT | 13 | 8 | 3 | NA | 8 | 3 | 6 | 1 | 14 | NA | 21 |
| RM8747 | O15:NT | 14 | 6 | 3 | NA | 13 | 4 | 13 | 1 | 7 | NA | NA |
| RM8748 | O73:NT | 14 | 6 | 3 | NA | 13 | 4 | 13 | 1 | 7 | NA | NA |

^1^NT, H-antigen nontypeable; ONT, O-antigen nontypeable.

^2^NA, non-amplified PCR product.
